# NF‐YB1‐YC12‐bHLH144 complex directly activates *Wx* to regulate grain quality in rice (*Oryza sativa* L.)

**DOI:** 10.1111/pbi.13048

**Published:** 2019-01-04

**Authors:** Babatunde Kazeem Bello, Yuxuan Hou, Juan Zhao, Guiai Jiao, Yawen Wu, Zhiyong Li, Yifeng Wang, Xiaohong Tong, Wei Wang, Wenya Yuan, Xiangjin Wei, Jian Zhang

**Affiliations:** ^1^ State Key Lab of Rice Biology China National Rice Research Institute Hangzhou China; ^2^ State Key Lab of Biocatalysis and Enzyme Engineering Hubei Collaborative Innovation Center for Green Transformation of Bio‐Resources Hubei Key Laboratory of Industrial Biotechnology College of Life Sciences Hubei University Wuhan China

**Keywords:** nuclear factor Y, rice (*Oryza sativa* L.), grain quality, *Wx* gene, G‐box, heterotrimer complex

## Abstract

Identification of seed development regulatory genes is the key for the genetic improvement in rice grain quality. NF‐Ys are the important transcription factors, but their roles in rice grain quality control and the underlying molecular mechanism remain largely unknown. Here, we report the functional characterization a rice NF‐Y heterotrimer complex NF‐YB1‐YC12‐bHLH144, which is formed by the binding of NF‐YB1 to NF‐YC12 and then bHLH144 in a sequential order. Knock‐out of each of the complex genes resulted in alteration of grain qualities in all the mutants as well as reduced grain size in *crnf‐yb1* and *crnf‐yc12*. RNA‐seq analysis identified 1496 genes that were commonly regulated by *NF‐YB1* and *NF‐YC12*, including the key granule‐bound starch synthase gene *Wx*. NF‐YC12 and bHLH144 maintain NF‐YB1 stability from the degradation mediated by ubiquitin/26S proteasome, while NF‐YB1 directly binds to the ‘G‐box’ domain of *Wx* promoter and activates *Wx* transcription, hence to regulate rice grain quality. Finally, we revealed a novel grain quality regulatory pathway controlled by NF‐YB1‐YC12‐bHLH144 complex, which has great potential for rice genetic improvement.

## Introduction

The modulation of transcription of most eukaryotic genes is coordinated through sequence‐specific binding of transcription factors to the promoter section situated upstream of the gene (Gelinas *et al*., 1985; Mantovani, 1998). CCAAT box, which is found between 80 and 300 bp from the transcription start site and may function in either direction, has been found to function as positive regulatory *cis*‐acting elements in various species including human (Martyn *et al*., 2016), mouse (Bernadt and Rizzino, 2003), yeast (McNabb *et al*., 1995) and filamentous fungus (Steidl *et al*., 1999). A diverse of transcription factors with different levels of specificity has been revealed to bind to different CCAAT boxes, (Dorn *et al*., 1987; Raymondjean *et al*., 1988), and each is thought to play a discrete role in DNA replication or gene expression (Santoro *et al*., 1988). The binding of CCAAT box by NF‐Ys (Nuclear Factor Y), which also named as HAP (Heme Activator Protein) or CBF (CCAAT box‐Binding Factor), is anticipated to be a significant mechanism required for transcription activation (Romier *et al*., 2003). NF‐Y family is comprised by three subunits NF‐YA, NF‐YB and NF‐YC. Protein structure analysis indicated that NF‐YA harbours two conserved α‐helix domains A1 and A2. The 20‐amino acids (AAs)‐long A1 domain locates in the N‐terminal side and is responsible for protein–protein interaction with NF‐YB and NF‐YC, whereas the C‐terminal locates A2 domain determines the binding specificity with CCAAT boxes (Laloum *et al*., 2013; Petroni *et al*., 2012). Like histone H2B and H2A, typical NF‐YB and NF‐YC subunits have a highly conserved histone fold domains (also called histone‐like domains) containing three α‐helices (α1, α2 and α3), and another α‐helix domain in the C‐terminal (αC; Frontini *et al*., 2004; Laloum *et al*., 2013). These domains confer the NF‐YB and NF‐YC subunits with both protein–protein and protein–DNA binding abilities. It has been demonstrated that NF‐Ys work as heterotrimer complexes to bind with the CCAAT‐containing regulatory elements. The complex forms in a sequential order by NF‐YB bind with NF‐YC, then the dimer complex bind with NF‐YA. NF‐YB and NF‐YC could not bind with NF‐YA without being in a dimer format (Petroni *et al*., 2012). For the NF‐Y complex in mammals, it has been clear that NF‐YB‐YC dimer binds to the sugar‐phosphate backbone of DNA flanking the CCAAT box in a manner similar to the H2A‐H2B‐DNA assembly. Such nonspecific DNA binding is supposed to stabilize the NF‐Y complex with DNA. Meanwhile, the NF‐YA part specifically binds to the CCAAT box by inserting into the minor groove of DNA to drive gene transcription (Oldfield *et al*., 2014).

In contrast to the conditions in mammals and yeasts that each NF‐Y subunit is encoded by a single gene, NF‐Y subunits in the plant kingdom contain multiple family members, which greatly expand the diversity of potential combinations of trimeric complexes (Laloum *et al*., 2013). For example, the 10 NF‐YAs, NF‐YBs and NF‐YCs in Arabidopsis could theoretically result in 1000 potential combinations, hence to extensively involve in diverse biological processes (Swain *et al*., 2016; Zhao *et al*., 2016). In other species such as soybean, maize and Medicago, *NF‐Ys* were also implicated in legume‐rhizobia symbiosis, Arbuscular mycorrhizal symbiosis and nitrogen starvation response (Zanetti *et al*., 2016). Rice (*Oryza sativa* L.) is one of the world's most important food crops as well as a model species for monocot molecular biology investigation (Itoh *et al*., 2005). In rice, various NF‐Y subunits are involved in specific developmental processes or responses to developmental signals, including seed nutrient accumulation, flowering time regulation, ABA response and chloroplast development (Lee *et al*., 2015; Li *et al*., 2015; Romier *et al*., 2003; Siefers *et al*., 2009; Sinha *et al*., 1995; Xu *et al*., 2016; Yamamoto *et al*., 2009). Rice genome contains 10 *NF‐YA*, 11 *NF‐YB* and 12 *NF‐YC* genes, among which *NF‐YBs* are the most well‐documented subunits (Gusmaroli *et al*., 2001; Petroni *et al*., 2012; Thirumurugan *et al*., 2008). *NF‐YB2/HAP3A*,* NF‐YB3/HAP3B* and *NF‐YB4/HAP3C* were found to be functional redundantly involved in the chloroplast biogenesis. Simultaneous suppressing of the three genes led to pale green leaves and degeneration of chloroplast formation (Miyoshi *et al*., 2003). *OsNF‐YB7/LEC1* is essential to rice vegetative and reproductive growth. *lec1* mutants were lethal, but the *LEC1* overexpression lines displayed erected leaves and defected panicles and spikelet (Ito *et al*., 2011; Zhang and Xue, 2013). *NF‐YB11/DTH8/Ghd8* is a master regulator of rice heading date, plant height and yield. Under long‐day condition, *NF‐YB11* suppresses the transcription of *Ehd1* and *Hd3a* to delay the rice heading date (Dai *et al*., 2012; Wei *et al*., 2010; Yan *et al*., 2011). *NF‐YB11* is also implicated in chloroplast biogenesis and carbon assimilation (Adachi *et al*., 2017; Feng *et al*., 2014).

*OsNF‐YB1* and *OsNF‐YB9* are endosperm‐specific genes that play a prominent role in preservation of endosperm cell development (Sun *et al*., 2014). *OsNF‐YB1* and *OsNF‐YB9* have been found to be co‐expressed with starch and storage protein synthesis related genes, suggesting their roles in accumulation of seed reserves (Yang *et al*., 2016). Recently, it has been demonstrated that *NF‐YB1* might impose multiple effects on grain cell proliferation, assimilate loading to endosperm and grain filling, and eventually regulate various aspects of seed development in rice (Bai *et al*., 2015; Sun *et al*., 2014; Xu *et al*., 2016). Despite that much understanding of NF‐YB1 function have been achieved so far, the detailed molecular mechanism underlying the NF‐YB1‐regulated seed development remains largely unknown, particularly its counterparts in forms of protein complex as well as the regulatory roles in the seed nutrient synthesis. In the current study, we demonstrated that NF‐YB1 interacts with NF‐YC12 and bHLH144 in a sequential order to form a heterotrimer complex. NF‐YC12 and bHLH144 maintain NF‐YB1 protein stability against ubiquitin/26S proteasome‐mediated degradation, while stable NF‐YB1 activates the transcription of the key granule‐bound starch synthase gene *Wx* by directly binding to the ‘G‐box’ domain of its promoter, hence to regulate the synthesis of amylose in rice.

## Results

### *NF‐YB1* is a seed‐specific gene and its protein locates in nucleus and cytoplasm

**Figure 1:**
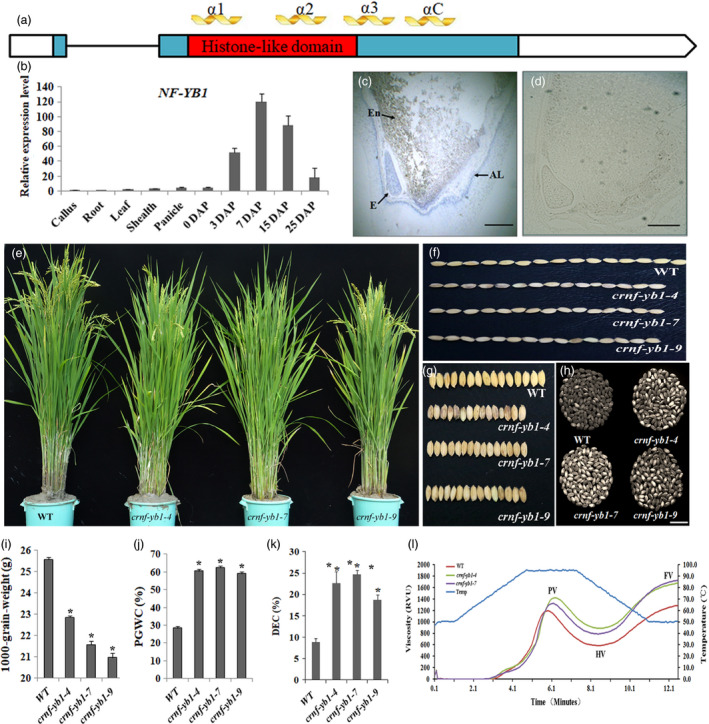
Spatial‐expression pattern of NF‐YB1 and phenotypical characterization of *crnf‐yb1s* and WT. (a) Schematic presentation of the *NF‐YB1* gene structures and conserved protein domains. Boxes: exons; blue box: coding sequences; red box: conserved HLD; line: intron; α1, α2, α13 and αC are four conserved α‐helix structures. (b) qRT‐PCR analysis of *NF‐YB1* transcription abundances in various tissues and stages. The expression level of callus was set as 1. (c) mRNA *in situ* hybridization analysis of *NF‐YB1* on 7 DAP seeds. E: embryo; En: endosperm; Al: aluerone layer. Scale bar = 1 mm. (d) Negative CK of (c) using sense probe for hybridization. (e) Plant morphology at grain‐filling stage. (f‐k) comparison of the seed length (f), width (g), chalkiness (h), 1000‐grain‐weight (i), percentage of Grain With Chalkiness (j) and Degree of Endosperm Chalkiness (k) of *crnf‐yb1s* and WT. Data are shown as Means ± SD of at least three biological replicates. *: *P* ≤ 0.05 by the Student's *t* test. (l) Pasting properties of seeds analysed by Rapid Visco Analyzer (RVA).

As indicated in the RiceGE (http://signal.salk.edu/cgi-bin/RiceGE5) database, *NF‐YB1* (*LOC_Os02g49410*) was specifically expressed in seeds. This 187 amino acids‐long protein contains a histone‐like domain (HLD) at position 33‐92 (https://www.ncbi.nlm.nih.gov/Structure/cdd/wrpsb.cgi; Marchlerbauer *et al*., 2017). In consistence with other NF‐YBs, 4 α‐helices structures were identified in NF‐YB1 protein, among which α1 and α2 were covered by HLD domain (Figure 1a). The qRT‐PCR result confirmed that *NF‐YB1* highly expressed in seeds, particularly in 7 DAP (Days After Pollination) seeds, in which the grains start to fill with starch and other nutrients (Figure 1b). mRNA *in situ* hybridization result showed that *NF‐YB1* majorly accumulated in embryo and alueron layer, but not in the endosperm of the 7 DAP seeds (Figure 1c). As shown in Figure S1a‐c, NF‐YB1 protein accumulated in nuclear and cytoplasm, which is consistent with the previous reports (E *et al*., 2018; Xu *et al*., 2016).

### Knock‐down of *NF‐YB1* reduced grain size and altered grain quality

Using CRISPR/Cas9 technique, we generated knock‐out/down lines of *NF‐YB1*. Sanger sequencing of the CRISPR/Cas9 target sites detected various types of deletion in the *crnf‐yb1‐4*,* crnf‐yb1‐7* and *crnf‐yb1‐9* respectively, which were believed to knock‐out the *NF‐YB1* by missing the start codon or shifting the open reading frame (Figure S2). The homozygous T_1_ mutants from the three representative lines were employed for a detailed phenotypic characterization. Not surprisingly, no visible phenotypes, such as plant height, flowering date and seed setting, were observed in the *crnf‐yb1* lines (Figure 1e and Table S1). However, *crnf‐yb1‐4*,* crnf‐yb1‐7* and *crnf‐yb1‐9* showed significant reduction in grain length and width, which consequently resulted in a significant reduction in grain size ranging from 82.02% to 89.41% (Figure 1f, g and i, Table S1). In addition, we observed higher chalkiness in *crnf‐yb1* mutants (Figure 1h), as both Percentage of Grain With Chalkiness (PGWC) and Degree of Endosperm Chalkiness (DEC) were significantly increased when compared with WT (*P* < 0.05; Figure 1j and k). A followed‐up phenotyping of the T_2_ plants of these three lines revealed consistent reduction in grain size in the mutants; hence we inferred that these phenotypes were ascribed to the knock‐out of *OsNF‐YB1* (Figure S3).

We determined the total starch, amylose, crude protein, crude fiber and lipid contents in the brown rice of WT and *crnf‐yb1s*. The mutants showed significant reduction in starch, amylose, lipid contents as well as elevated crude protein contents when compared to the WT (*P* < 0.05; Table 1).

**Table 1:** Nutrition content assays of *crnf‐yb1s*,* crnf‐yc12s* and *crbhlh144s*

	Starch (%)	Amylose (%)	Protein (%)	Crude fiber (%)	Lipid (%)
WT‐1	69.61 ± 1.013	18.61 ± 1.260	10.43 ± 0.003	1.81 ± 0.001	2.53 ± 0.001
*crnf‐yb1‐4*	57.76 ± 4.801*	15.15 ± 0.294**	12.90 ± 0.010**	1.74 ± 0.001*	2.14 ± 0.007*
*crnf‐yb1‐7*	60.39 ± 3.164**	16.85 ± 1.514**	13.63 ± 0.003**	1.59 ± 0.002*	2.11 ± 0.005**
*crnf‐yb1‐9*	58.16 ± 1.874*	15.81 ± 0.592*	14.50 ± 0.001**	1.61 ± 0.002*	2.16 ± 0.001**
*crnf‐yc12‐7*	56.30 ± 19.765*	14.21 ± 0.007*	11.58 ± 0.062*	1.65 ± 0.001**	2.06 ± 0.008*
*crnf‐yc12‐11*	56.30 ± 19.765*	14.21 ± 0.007*	11.58 ± 0.062*	1.65 ± 0.001**	2.06 ± 0.008*
*crnf‐yc12‐14*	55.13 ± 1.182**	13.96 ± 0.363*	12.49 ± 0.909*	1.52 ± 0.001**	2.69 ± 0.002*
WT‐2	68.93 ± 1.724	19.20 ± 1.221	9.93 ± 0.003	1.95 ± 0.002	2.77 ± 0.003
*crbhlh144‐2*	54.38 ± 1.463*	18.27 ± 0.470*	11.11 ± 0.003*	1.72 ± 0.002*	2.32 ± 0.002*
*crbhlh144‐3*	54.96 ± 2.492**	17.88 ± 0.549*	11.03 ± 0.001*	1.74 ± 0.002*	2.45 ± 0.003*
*crbhlh144‐4*	58.66 ± 1.450*	18.59 ± 0.436	11.76 ± 0.001*	1.65 ± 0.001*	2.31 ± 0.003*

Brown seeds were used for the assays. These data are presented as the Means ± SD of at least three biological replicates. The asterisks represent significant difference between the WT and mutants as determined by the Student's *t* test, the single asterisk indicates *P* ≤ 0.05, double asterisks indicate *P* ≤ 0.01. WT‐1 is the control for *crnf‐yb1s* and *crnf‐yc12s*, WT‐2 is the control for *crbhlh144s*.

Rapid Visco Analyzer (RVA) was employed to analyse the pasting properties of the starch of *crnf‐yb1‐4, crnf‐yb1‐7* and WT. We did not include the *crnf‐yb1‐9* sample due to the unavailability of enough seeds. *crnf‐yb1* mutants exhibited higher values of viscosity parameters including peak viscosity (PV), hot viscosity (HV) and final viscosity (FV; Figure 1l). Furthermore, we analysed the thermal properties of the mutants and WT using differential scanning calorimetry (DSC). In this assay, onset temperature is used to indicate the starting temperature for melting of the amylopectin crystallities, while gelatinization enthalpy reflects the required heat energies for amylopectin melting. Interestingly, we found the onset, peak and end gelatinization temperatures as well as the gelatinization enthalpy were all significantly decreased in *crnf‐yb1*s (Table S2). The results above implied that *NF‐YB1* may also affect the starch pasting and alkaline gelatinization properties.

We further generated *NF‐YB1* overexpression lines, multiple independent lines showed substantial enhanced expression level, but no significant phenotypes were observed in seed size (Figure S4). This phenomenon indicates that NF‐YB1 may work as a complex with other proteins; elevating the level of NF‐YB1 alone imposes no effect on the protein complex activity.

### NF‐YB1, NF‐YC12 and bHLH144 forms a hereotrimer complex in a sequential order

**Figure 2:**
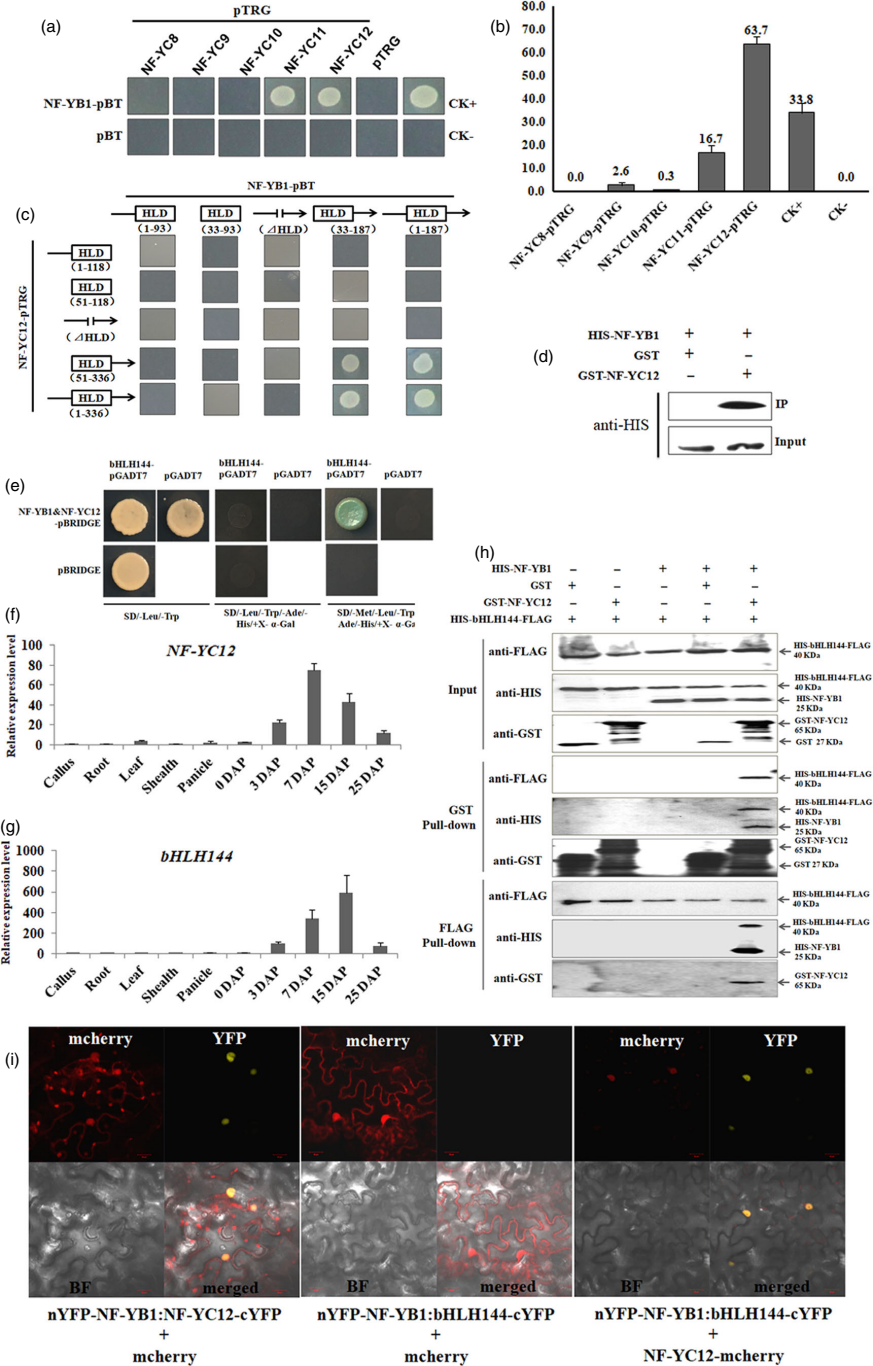
Protein–protein interaction analysis of NF‐YB1, NF‐YC12 and bHLH144. (a) Bacterial‐two‐hybrid analysis of NF‐YB1 with NF‐YCs. LGF2‐pBT and Gal11‐pTRG were used as CK+, pBT and pTRG empty vector was used as CK‐. (b) Quantification of the interaction intensities between NF‐YB1‐pTRG and NF‐YCs‐pBT. The values on the Y axis represent the ratio (%) between colony numbers grow out on medium M9/‐HIS/+Cm/+Ter/+Strep and M9/+Cm/+Ter. (c) Interaction test of truncated NF‐YB1 and NF‐YC12 by bacterial‐two‐hybrid. The numbers underneath indicate the positions of the truncated regions on the protein. The position of HLD on NF‐YB1 is 31‐93, and on NF‐YC12 is 53‐115. ⊿HLD indicates HLD was truncated out. (d) *in vitro* GST pull‐down assay of HIS‐NF‐YB1 and GST‐NF‐YC12. (e) Y3H analysis of NF‐YB1, NF‐YC12 and bHLH144. (f‐g) qRT‐PCR analysis of *NF‐YC12* and *bHLH144*. (h) *in vitro* GST pull‐down assay of HIS‐NF‐YB1, GST‐NF‐YC12 and HIS‐FLAG‐bHLH144. (i) BiFC assay of NF‐YB1, NF‐YC12 and bHLH144 interactions. Two tested proteins were constructed in vector pDOE‐BiFC, which generates YFP fluorescence when the two bind with each other. The third protein or empty control was fused with mcherry to generate red fluorescence. BF: bright field. Scale bar = 20 μm.

To find out the NF‐YC counterparts of NF‐YB1, we conducted a bacterial‐two‐hybrid assay between NF‐YB1 and other five seed‐specific NF‐YC proteins, namely NF‐YC8 (LOC_Os01g01290), NF‐YC9 (LOC_Os01g24460), NF‐YC10 (LOC_Os01g39850), NF‐YC11 (LOC_Os05g23910) and NF‐YC12 (LOC_Os10g11580). NF‐YB1 could physically bind with NF‐YC11 and NF‐YC12 in *Escherichia coli*, but not or very weakly interact with the other three NF‐YCs (Figure 2a). The interaction intensity of NF‐YB1‐NF‐YC12 (63.7%) almost doubled that of the positive control (33.8%), suggesting a very strong interaction (Figure 2b). Given that both proteins contain a conserved histone‐like domain (HLD) in the middle of the protein, we constructed different truncated versions of NF‐YB1 and NF‐YC12 to test their interactions. As shown in Figure 2c, for both NF‐YB1 and NF‐YC12, the absence of 5′ upstream regions of HLD did not affect the protein–protein interaction. However, the binding was compromised when either HLD domain or its 3′ downstream regions were truncated out. Hence, the indication is that the HLD domain is necessary, but insufficient for the NF‐YB1‐NF‐YC12 interaction, some unknown elements in the 3′ regions are essential for their bindings. We also conducted *in vitro* GST pull‐down assays to validate the interaction of NF‐YB1 and NF‐YC12. Indeed, HIS‐NF‐YB1 was pulled down with GST‐NF‐YC12, whereas GST tag alone was not able to pull‐down HIS‐NF‐YB1 (Figure 2d).

Yeast‐three‐hybrid (Y3H) was employed to further screen for the counterparts of the NF‐YB1‐YC12 heterodimer. In the Y3H system, NF‐YB1 was fused with GAL4 DNA binding domain, while NF‐YC12 was driven under a *Met25* promoter which is conditionally expressed in the absence of methionine. Given that NF‐YA is the mostly identified components of NF‐Y complex in previous studies, we firstly checked the interactions between NF‐YB1‐YC12 heterodimer and three seed‐specific NF‐YAs, namely NF‐YA1 (LOC_Os03g07880), NF‐YA5 (LOC_Os07g41720) and NF‐YA8 (LOC_Os10g25850). Unfortunately, all the three tested NF‐YAs displayed autoactivations in the Y3H experimental system (Figure S5). Thus, we moved to screen a seed‐derived prey library using NF‐YB1‐YC12 as the bait, and finally detected an interactive protein bHLH144 (LOC_Os04g35010)*,* which is encoded by a seed‐specifically transcribed gene as its counterpart genes *NF‐YB1* and *NF‐YC12* (Figure 2e, f and g). Interestingly, the interaction between NF‐YB1 and bHLH144 is valid only on the SD/‐Met/‐Leu/‐Trp/‐Ade/‐His/+X‐α‐Gal medium, in which the drop‐out of metheonine drove the expression of NF‐YC12 under *Met25* promoter, whereas NF‐YB1 alone did not bind to bHLH144 on the SD/‐Leu/‐Trp/‐Ade/‐His/+X‐α‐Gal medium, in which NF‐YC12 was absent, as the supplemented metheonine in the medium could inhibit the expression of NF‐YC12. Therefore, the Y3H results suggested that the binding of NF‐YB1‐YC12 is pre‐required for the formation of the heterotrimer complex (Figure 2e). To verify the Y3H result, we performed the pull‐down assay with glutathione agarose beads and FLAG agarose beads respectively (Figure 2h). In the GST pull‐down assay, GST‐NF‐YC12 could only pull‐down the HIS‐bHLH144‐FLAG only when HIS‐NF‐YB1 was present. FLAG pull‐down assay obtained a similar result. Neither GST‐NF‐YC12 nor HIS‐NF‐YB1 alone was pulled down by HIS‐bHLH144‐FLAG, but both were pulled down by HIS‐bHLH144‐FLAG in the reaction containing the three proteins. Such a protein–protein interaction pattern was again validated *in planta* by bimolecular fluorescence complementation (BiFC) assay. The results showed that NF‐YB1 interacted with NF‐YC12 to form a heterodimer with YFP fluorescence. However, NF‐YB1 interacted with bHLH144 to show YFP fluorescence only when NF‐YC12‐mcherry was presented, and all the three proteins were co‐localized in nuclear with overlapped fluorescence (Figure 2i). Taken together, the above results suggested that the NF‐YB1‐YC12‐bHLH144 heterotrimer complex is formed in a sequential order by NF‐YB1 bind with NF‐YC12, and then with bHLH144, while bHLH144 alone does not interact with NF‐YB1 or NF‐YC12.

### *crnf‐yc12* and *crbhlh144* exhibited similar phenotype to *crnf‐yb1* in seed development

**Figure 3:**
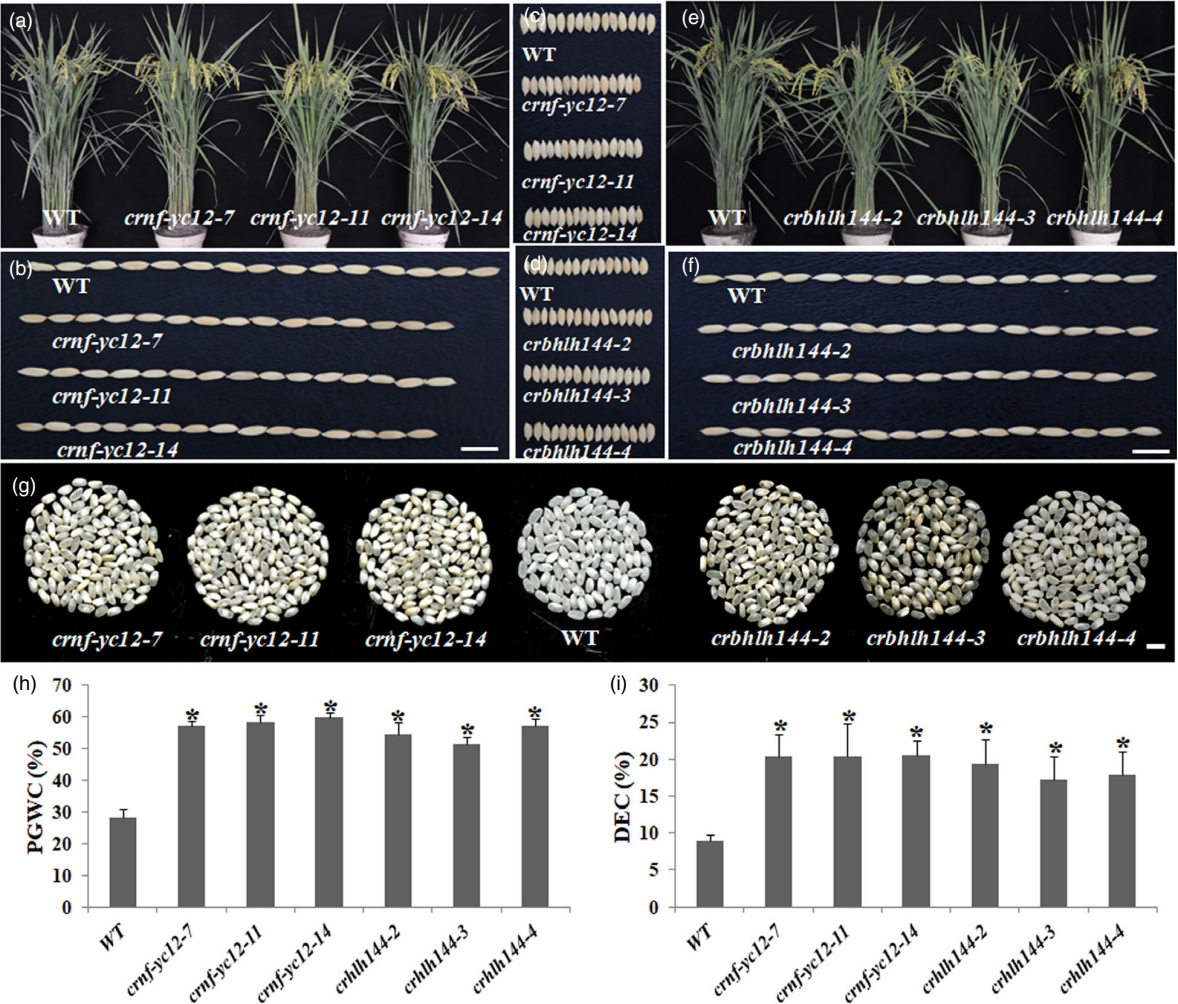
Phenotypical characterization of *crnf‐yc12s, crbhlh144s* and WT. (a) Plant morphology of *crnf‐yc12s* and WT at grain‐filling stage. (b‐c) comparison of the seed length (b) and width (c) of *crnf‐yc12s* with WT. (d‐f) comparison of the seed width (d), length (f) and plant morphology of *crnf‐yc12s* and WT at grain‐filling stage (e). (g) Comparison of chalkiness of *crnf‐yc12s* and *crbhlh144s* with WT. Percentage of Grain With Chalkiness (h) and Degree of Endosperm Chalkiness (i) of *crnf‐yc12s, crbhlh144s* and WT. Data are shown as Means ± SD of at least three biological replicates. *: *P* ≤ 0.05 by the Student's *t* test.

We generated mutants of the *NF‐YC12* and *bHLH1144* using CRISPR/Cas9 technique. In T_1_ generation, three representative homozygous mutant lines of *NF‐YC12* (*crnf‐yc12‐7, crnf‐yc12‐11* and *crnf‐yc12‐14*) and three of *bHLH144* (*crbhlh144‐2, crbhlh144‐3* and *crbhlh144‐4*), which were confirmed with shifted open reading frames, were morphologically characterized along with the WT (Figure S2). Similar to *crnf‐yb1s*,* crnf‐yc12s* and *crbhlh144s* showed almost identical morphology with the WT in vegetative growth (Figure 3a and e; Table S1). However, there were around 20% reduction in the 1000‐grain‐weight in *crnf‐yc12s,* when compared with the WT (Figure 3b, c and Table S1). In contrast, *bHLH144* may not be functionally related to grain size control as the three mutant lines displayed similar size as the WT (*P* > 0.05; Figure 3d, f and Table S1). *crnf‐yc12s* and *crbhlh144s* all had higher PGWC and DEC (Figure 3g, h and i). The grain quality assay of *crnf‐yc12s* and *crbhlh144s* obtained similar results as *crnf‐yb1s*. The mutants showed significant reduction in starch, amylose, lipid contents as well as elevated crude protein contents when compared to the WT (*P *< 0.05), which exactly phenocopied *crnf‐yb1s* (Table 1).

### *NF‐YB1* and *NF‐YC12* co‐regulate transcription of genes involves in starch biosynthesis

RNA‐sequencing experiments were performed on the 7 DAP seeds of *crnf‐yb1‐4*,* crnf‐yc12‐11* along with the WT to investigate the potential downstream genes. In *crnf‐yb1‐4*, 574 up‐regulated genes and 1618 down‐regulated genes were identified (|log_2_Ratio| ≥1; FDR <0.001). We also found 480 up‐regulated and 1493 down‐regulated genes in *crnf‐yc12‐11*. By merging the data together, we finally identified 310 and 1186 DEGs that were commonly up‐regulated and down‐regulated respectively in both *crnf‐yb1* and *crnf‐yc12* plants (Table S4, Figure S6). KEGG pathway enrichment analysis of the common DEGs revealed that many metabolism pathways such as starch and sucrose metabolism, carbon metabolism, photosynthesis were overpresented, which is consistent with the observed phenotype in grain nutrient accumulation and quality (Figure S7).

**Figure 4:**
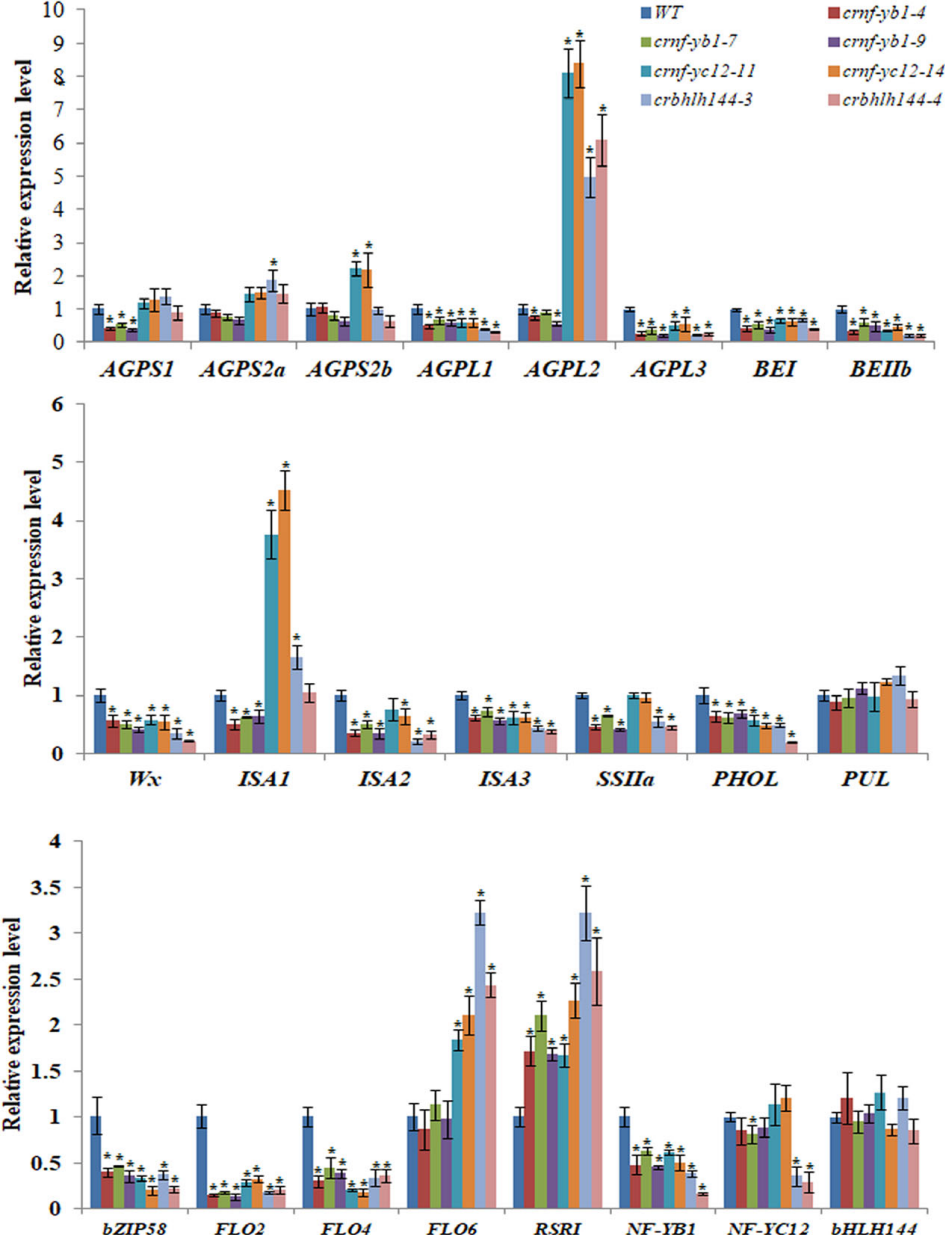
qRT‐PCR analysis of the transcriptional abundances of starch biosynthesis‐related DEGs and master regulators in 7 DAP seeds of *crnf‐yb1s, crnf‐yc12s, crbhlh144s* and WT. The asterisk represents significant difference with the WT at *P* ≤ 0.05 as determined by the Student's *t* test.

The Differentially Expressed Genes (DEGs) covered a large number of starch synthesis genes, and other nutrient synthesis genes. We performed qRT‐PCR analysis of the DEGs as well as some reported rice seed development regulator genes in *crnf‐yb1*,* crnf‐yc12* and *crbhlh144* with multiple biological replicates (Figure 4). A number of the starch synthesis genes, such as *AGPL1* (*ADP‐glucose pyrophosphorylase large subunit 1*;* LOC_Os03g52460*), *AGPL3* (*LOC_Os05g50380*) and *Wx* (*LOC_Os06g04200*) were commonly down‐regulated in the mutants of all the three genes, indicating that these DEGs are under common pathways regulated by the NF‐YB1‐YC12‐bHLH144 complex. Meanwhile, *AGPS1* (*ADP‐glucose pyrophosphorylase small subunit 1*) (*LOC_Os09g12660*), *AGPS2b* (*ADP‐glucose pyrophosphorylase small subunit 2b*) (*LOC_Os08g25734*), *SSIIa* (*Starch Synthesase II a*) (*LOC_Os06g12450*) were only differentially expressed in one or two of the three mutants, suggesting that *NF‐YB1*,* NF‐YC12* and *bHLH144* have their independent regulatory pathways in seed development. Additionally, the expression of chalkiness controlling genes *FLO2* (*Floury endosperm 2*) (*LOC_Os04g55230*) and *FLO4* (*Floury endosperm 2*) (*LOC_Os05g33570*), grain nutrient synthesis regulators *RSR1* (*Rice Starch Regulator 1*) (*LOC_Os05g03040*) and *bZIP58* (*LOC_Os07g08420*) were also differentially expressed in the mutants (Figure 4).

### NF‐YB1 binds to the G‐box of *Wx* promoter to activate its transcription

**Figure 5:**
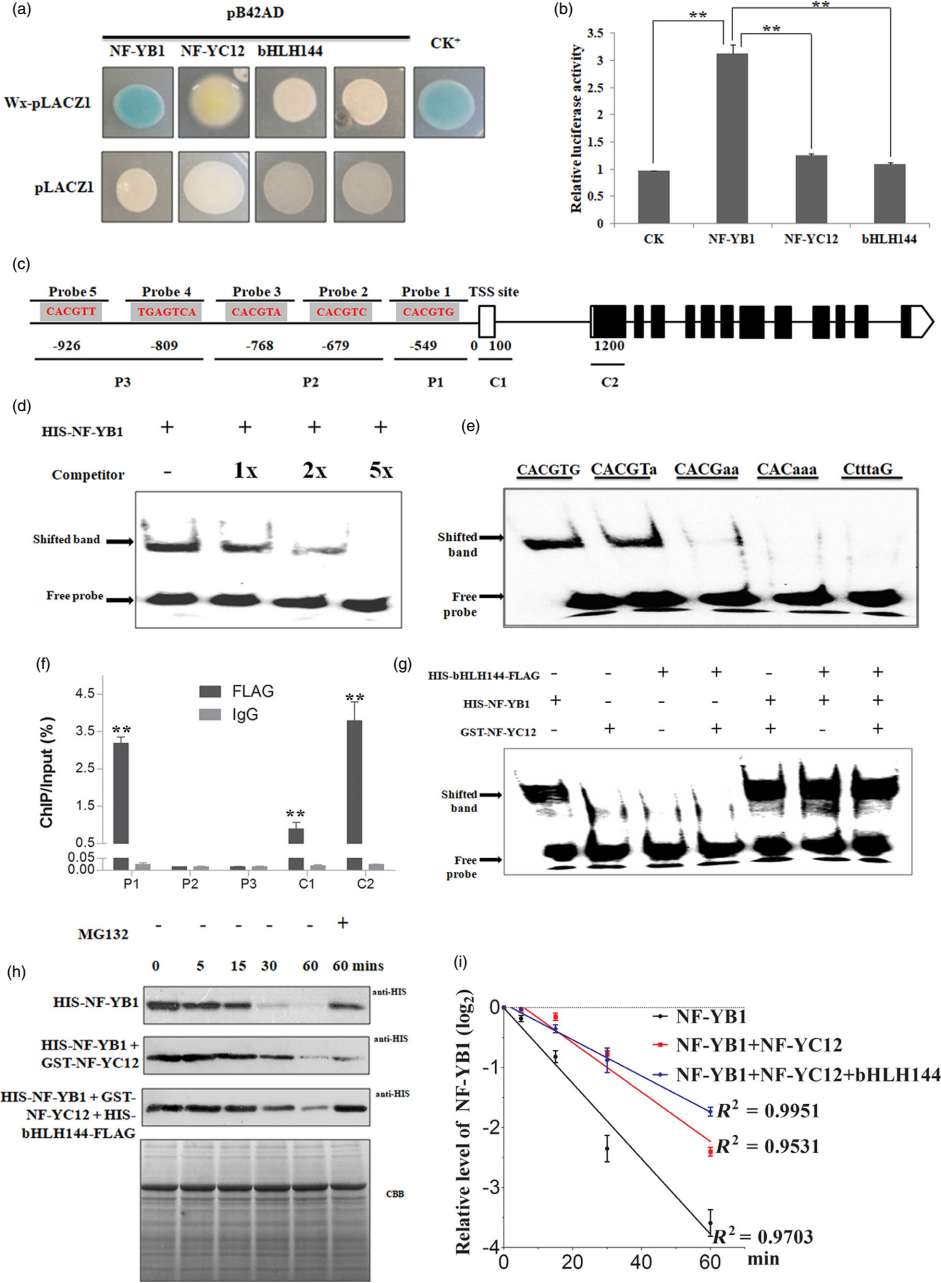
NF‐YB1 directly binds to the G‐box of *Wx* promoter and activates its transcription. (a) Y1H assay of NF‐YB1, NF‐YC12 and bHLH144 to the promoter of *Wx*. NF‐YB1‐pB42AD and SUT4‐pLACZ1 were used as CK+. (b) LUC transient transcriptional activity assay in rice protoplast. Reporter: *proWx:LUC*; Effectors: *pro35S:NF‐YB1:tNOS*,* pro35S:NF‐YC12:tNOS* and *pro35S:bHLH144:tNOS*. Values are mean ± SD with biological triplicates. **: *P* < 0.01 by the Student's *t* test. (c) Probe positions on *Wx* promoter. Transcription Starting Site (TSS) was set as 0; Numbers indicate the distances (bps) of probes to the TSS. Red letters indicate the core element sequences in the position. (d) EMSA assay to show NF‐YB1 binds with the probe 1 on the promoter of *Wx*. (e) EMSA assay showing the binding of NF‐YB1 with mutated G‐box probe 1. (f) ChIP qRT‐PCR assay showing the NF‐YB1 bind to *Wx* promoter regions. Values are mean ± SD with biological triplicates. The enrichment values were normalized to Input. IgG immunoprecipitated DNA was used as a CK. **: *P* < 0.01 in comparison with the IgG mock samples. (g) EMSA assay showing the binding intensities of NF‐YB1 with probe 1 in the presence of NF‐YC12 and bHLH144. (h) Degradation assay of HIS‐NF‐YB1 under the absence or presence of GST‐NF‐YC12 and/or HIS‐bHLH144‐FLAG. Equal starting amount of the total proteins were used for the degradation as indicated by the Commassie blue staining. (i) Degradation curve of HIS‐NF‐YB1deduced from the result of (h).

*Wx* has been reported as a master regulator of amylose biosynthesis (Tian *et al*., 2009; Wang *et al*., 1995). The reduced amylose content as well as down‐regulated *Wx* level in the mutants implied that *Wx* is a direct target gene of them. The hypothesis was firstly tested by yeast‐one‐hybrid (Y1H) experiment. The results showed that NF‐YB1, but not NF‐YC12 and bHLH144 activated the *LacZ* expression (Figure 5a). To verify the NF‐YB1‐mediated activation on *Wx*, we also performed luciferase (LUC) transient transcriptional activity assay in protoplasts. Strong activation of LUC were detected in *pro35S:NF‐YB1:tNOS*, though the other two proteins also showed weak but significant activations of LUC when compared with the negative control, suggesting that NF‐YB1 activates the *Wx* transcription *in vivo* (Figure 5b).

Subsequently, we conducted electrophoresis mobility shift assay (EMSA) to test the binding *in vitro*. In the 1 kb *Wx* promoter region, we found four G‐box, one GCN box, but no typical CCAAT box elements by using the online tool PlantCARE (Plant Cis‐Acting Regulatory Elements; Lescot *et al*., 2002; Figure 5c). As a result, the shift speed of only probe 1, which represented the closest (549 bp) G‐box to transcription starting site (TSS), was retarded by HIS‐NF‐YB1 protein (Figure S8), and the shifted band signal was substantially weakened with the application of unlabeled, competitive probe 1, suggesting binding is highly specific (Figure 5d). It also appeared that probes with mutated G‐box lost the binding ability with NF‐YB1 (Figure 5e), therefore the G‐box in probe 1 is a core binding site for NF‐YB1. Such a binding pattern of NF‐YB1 on *Wx* promoter was further confirmed by our ChIP‐qPCR experiment. We found significant enrichment of P1 region, which covered the probe 1 in EMSA assay, but no signal in P2 and P3 regions, which represented the negative binding regions in EMSA assay (Figure 5f). Hence, we concluded that NF‐YB1 binds to the closest G‐box to the TSS in *Wx* promoter *in vivo*.

As a master regulator of amylose biosynthesis, *Wx* has been used as a target site of genome editing for the breeding of sticky rice varieties with very low amylose content (Zhang *et al*., 2018). We introduced *wx* mutations into Ningjin 7, an elite *japonica* cultivar and Huazhan, an *elite indica* restore line for hybrid rice breeding in China. Homozygous mutation of *Wx* in both backgrounds resulted in complete chakiness in the endosperm, demonstrating the great effect of *Wx* on rice grain quality control (Figure S9).

### NF‐YC12 and bHLH144 enhances NF‐YB1 stability

We performed EMSA assay to investigate the effect of NF‐YC12 and bHLH144 on the NF‐YB1 binding ability to *Wx* promoter (Figure 5g). In contrast to the strong binding ability of HIS‐NF‐YB1, neither GST‐NF‐YC12 nor HIS‐FLAG‐bHLH144 could bind to the *Wx* promoter probe 1. The additions of GST‐NF‐YC12 or HIS‐FLAG‐bHLH144 or both also did not alter the binding intensity of HIS‐NF‐YB1 to the *Wx* promoter, implying that the interaction with NF‐YC12 or bHLH144 does not affect the NF‐YB1 DNA binding ability.

Previous studies reported that complex components may affect the protein stability of each other. We were intrigued to conduct a cell‐free degradation assay of HIS‐NF‐YB1 by incubating the protein with total protein extracts from 10 DAGs (Days After Germination) WT seedlings (Figure 5h). As a result, the half‐life of HIS‐NF‐YB1 is around 15 min. GST‐NF‐YC12 dramatically stabilized HIS‐NF‐YB1 with a half‐life of 30 min. The best stability of HIS‐NF‐YB1 was achieved in a status of NF‐YB1‐YC12‐bHLH144 heterotrimer complex, as the HIS‐NF‐YB1 half‐life reached 40 min. Moreover, the application of MG132, an inhibitor of 26S proteasome degradation system, significantly suppressed the HIS‐NF‐YB1 degradation. Therefore, the above results suggested that NF‐YB1 is under the 26S proteasome‐mediated degradation, while the presence of NF‐YC12 and bHLH144 helps NF‐YB1 to maintain a stable protein status, and possibly enhances the NF‐YB1‐imposed transactivation of *Wx*.

**Figure 6:**
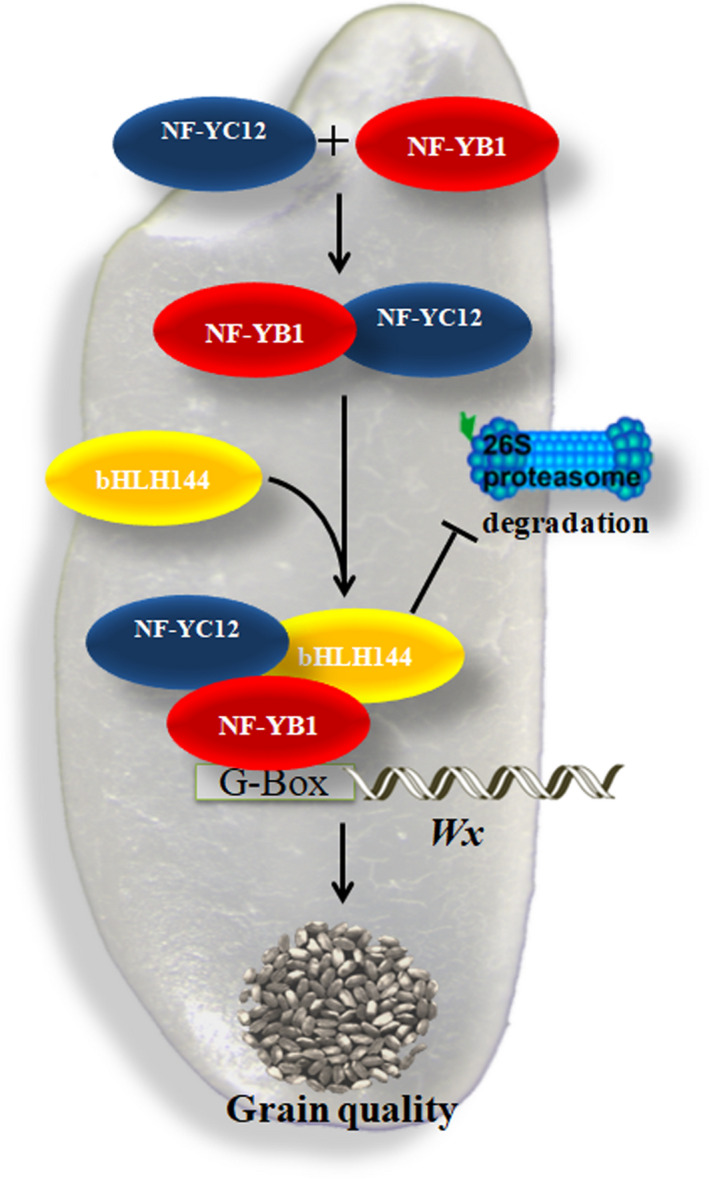
A working model for NF‐YB1‐YC12‐bHLH144 complex regulating starch synthesis in rice seeds. NF‐YB1 binds to NF‐YC12 to form a dimer, and then bind with bHLH144 to form a heterotrimer. The trimer complex protects NF‐YB1 against the ubiquitin/26S proteasome‐mediated degradation. Stable NF‐YB1 activates the transcription of the key granule‐bound starch synthase gene *Wx* by directly binding to the “G‐box” domain of its promoter, hence to regulate the starch synthesis.

Taken together, the current study demonstrated a novel transcriptional regulation mechanism of NF‐Ys in rice starch synthesis (Figure 6). During the rice seed development, NF‐YC12 and bHLH144 sequentially bind with the key regulator NF‐YB1 to prevent its degradation by the ubiquitin/26S proteasome pathway. Stable NF‐YB1 activates the transcription of the key granule‐bound starch synthase gene *Wx* by directly binding to the ‘G‐box’ domain of its promoter, hence to regulate the starch synthesis and grain quality.

## Discussion

### Comprehensive effects of NF‐YB1 on seed development

During the process of our *NF‐YB1* project, at least four independent labs reported their works on *NF‐YB1* with similar biological functions (Bai *et al*., 2015; E *et al*., 2018; Sun *et al*., 2014; Xu *et al*., 2016). In consistence with this study, RNAi or CRISPR/Cas9 knock‐out lines of *NF‐YB1* had smaller seeds and chalky endosperms, when compared with the WT. However, divergent mechanisms were proposed to support its function in rice endosperm development. Sun *et al*. (2014) found that repression of *OsNF‐YB1* resulted in differential expression of the genes in cell cycle pathway, and reduced endosperm cell numbers disintegrated with the development of abnormal seeds in *OsNF‐YB1* RNAi plants (Sun *et al*., 2014). Bai *et al*. (2015) emphasized the key role of *NF‐YB1* in modulating the expression of sucrose transporters in aleurone to enhance sugar loading to the endosperm (Bai *et al*., 2015). As described in a very recent publication, by forming a heterotrimer complex with NF‐YC and OsERF115, NF‐YB1 promote the binding of OsERF115 to GCC box of the downstream gene promoters, hence to regulate rice grain filling and endosperm development (Xu *et al*., 2016). In this study, we revealed that *NF‐YB1* regulates various aspects of grain quality and nutrient synthesis, in addition to the grain size and chalkiness. *crnf‐yb1* plants showed significantly lower starch, amylose, lipid contents, elevated crude protein contents as well as altered starch pasting and alkaline gelatinization properties, when compared with the WT. In line with this observation, our RNA‐seq and qRT‐PCR experiments identified a list of DEGs which are functionally related to starch synthesis and seed development. Notably, we found that the key amylose synthesis gene *Wx* was over twofold down‐regulated (Figure 4). *Wx* has been well‐known as a determinant gene controlling rice amylose content, gel consistency and gelatinization temperature (Tian *et al*., 2009; Wan *et al*., 2007; Wang *et al*., 1995). Suppression of *Wx* in *indica* or *japonica* backgrounds led to significantly lower amylose content, smaller seed size accompanied with changes in the content of glucose, sucrose and other cell‐wall polysaccharides (Figure S9; Chen *et al*., 2006; Zhang *et al*., 2012). Moreover, we provided multiple lines of evidence to demonstrate that NF‐YB1 transactivated the *Wx* expression in yeast, *in vitro* and *in vivo* (Figure 5), indicating *Wx* is a direct target of NF‐YB1 transcription factor. To the best of our knowledge, NF‐YB1 is the first reported direct regulator of *Wx*. Together with the previous reports, we speculate that *NF‐YB1* is a master regulator imposing comprehensive effects on seed development *via* controlling cell proliferation, assimilate transportation and nutrient biosynthesis.

### NF‐YB directly activates *Wx via* binding to the G‐box in promoter

Previously, it is believed that NF‐YA drives the target gene transcription by specifically binding with the conserved CCAAT box on the promoter, whereas NF‐YB and NF‐YC possesses no transactivation activities (Liu and Howell, 2010; Zhao *et al*., 2016). However, the function of each NF‐Y subunit may not be strictly conserved in plants. A recent publication reported a systematical analysis of the transactivation activity of rice NF‐Y subunits, and found that NF‐YB1, NF‐YB9, NF‐YC11 and NF‐YC12 showed no transcriptional activation, whereas NF‐YC8, NF‐YC9 and NF‐YC10 did (E *et al*., 2018). In the case of NF‐YB1, a controversial case also reported that it directly attached to the CCAAT box of several sucrose transporter gene promoters and activated their transcription *in vivo*, suggesting the functional overlapping of NF‐YB and NF‐YA in rice (Bai *et al*., 2015).

For the non‐canonical NF‐Y complexes in which NF‐YA is absent, the binding *cis*‐elements of the target genes may be determined by the non‐NF‐Y subunits (Kumimoto *et al*., 2010; Yamamoto *et al*., 2009). One well‐documented example is the Arabidopsis NF‐YB9‐C2‐bZIP67 complex, which could directly bind to the conserved bZIP binding site ABA‐responsive elements through bZIP67 (Yamamoto *et al*., 2009). Using ChIP‐seq technique, Xu *et al*. (2016) revealed that NF‐YB1 binding sites were enriched with conserved ERF binding elements GCC box. However, the binding is indirect, and the recruitment of NF‐YB1 to GCC sites was possibly mediated by its interactive protein OsERF115 (Xu *et al*., 2016). In the current study, for the NF‐YB1‐YC12‐bHLH144 complex, it is NF‐YB1, instead of the non‐NF‐Y member bHLH144, binds to and activates its downstream gene *Wx* (Figure 5a and c). Moreover, EMSA result demonstrated that NF‐YB1 directly and specifically binds to the G‐box of *Wx* promoter, in an independent manner of the presence of NF‐YC12 and/or bHLH144, indicating a novel mechanism of NF‐YB‐mediated gene transcriptional regulation. The G‐box has been known as a ubiquitous DNA element in various plant gene promoters, and functionally involves in responses to phytohormones and environmental stimuli (Menkens and Al, 1995). Although nearly all the identified GBFs (G‐box Binding Factors) are bZIPs, a few bHLHs including PIF3 (Phytohormone Interacting Factor 3) and TFHP‐1 (Transcription Factor bHLH Protein‐1) were found to be able to bind with G‐boxes (Kawaoka *et al*., 1994; Ni *et al*., 1998). We have excluded the involvement of bHLH144 in the binding of NF‐YB1 with G‐box on *Wx*, yet it remains to be explored that whether other bZIPs or bHLHs potentially participated in this event or not.

### NF‐YB1‐YC12‐bHLH144 works as a complex

In yeast and human, NF‐Y heterotrimer complex is comprised by a NF‐YB, a NF‐YC and a NF‐YA subunit. Such typical NF‐Y heterotrimers have also been identified in plants. For examples, Sato *et al*. (2014) reported the identification of Arabidopsis NF‐YA1‐B6‐C10 complex and their functions in heat and drought responses (Sato *et al*., 2014). Additionally, several recent studies have reported that NF‐YB‐YC interacted with other proteins, instead of NF‐YAs. For examples, CONSTANS (CO), which is a key flowering time regulator in Arabidopsis, was found to form complexes by interacting with multiple NF‐YC subunits (C1, C3, C4 and C9) and NF‐YB subunits (B2 and B3; Kumimoto *et al*., 2010). Other proteins, such as bZIP28, bZIP67, RGA (Repressor of GA) and RGL2 (RGA‐Like 2) were also found to interact with NF‐Ys to involve in biological processes in Arabidopsis (Hou *et al*., 2014; Huang *et al*., 2015; Liu and Howell, 2010; Yamamoto *et al*., 2009).

Here, we revealed the sequential interactions of NF‐YB1, NF‐YC12 and bHLH144 by Y3H, *in vitro* pull‐down assay and BiFC *in planta*, suggesting that NF‐YB1, NF‐YC12 and bHLH144 work as a heretortrimer complex. This hypothesis is supported by several other indirect evidence: (i). the three transcription factors are co‐expressed with a seed‐specific expression pattern, and co‐localized in nuclear; (ii). NF‐YB1 and NF‐YC12 shared a large number of DEGs; and (iii). *nf‐yb1, nf‐yc12* and *bhlh144* mutants phenocopied each other in seed development, though *bhlh144* did not show significant differences in grain size. Such a finding is consistent with the previous report that NF‐YB and NF‐YC could only interact with other proteins, like NF‐YA, by forming a heterodimer (Petroni *et al*., 2012). Nevertheless, few literatures also described that NF‐YB1 could directly bind to OsMADS18 and OsERF115 without the assistance of other NF‐YCs (Masiero *et al*., 2002; Xu *et al*., 2016). It should be noted that these reported interactions were only verified using Y2H experiments which is highly risky to yield potential false positive result. Although no rice NF‐YAs interacting with NF‐YB1‐YC12 dimer were detected in our Y3H screening, we could not exclude the participation of NF‐YAs, especially seed‐specific NF‐YAs, in forming the complex. Given the autoactivities of the NF‐YAs in yeast, alternative methods such as *in vitro* pull‐down or Co‐IP will be employed to test this hypothesis in our future work.

A cytoplasm‐nuclear shuttling model has been proposed to explain the effect of NF‐YC on NF‐YB. In this model, NF‐YB is originally located in cytoplasm, where it assembled with NF‐YC and transported to nuclear to form heterotrimer with the third member, either NF‐YAs or other proteins (Hackenberg *et al*., 2012; Zhao *et al*., 2016). Indeed, Xu *et al*. (2016) demonstrated that NF‐YB1 was specifically targeted from cytoplasm to nuclear by interacting with NF‐YC12 and other NF‐YCs (Xu *et al*., 2016). Based on our results, it seemed that NF‐YC12 and bHLH144 imposed no effect on the DNA binding capacity of NF‐YB1 to the *Wx* promoter (Figure 5g). However, our cell‐free degradation assay found that NF‐YB1 was under the degradation of ubiquitin/26S proteasome pathway, while binding with NF‐YC12 and bHLH144 greatly enhanced the NF‐YB1 stability *in vitro*, hence maintained the NF‐YB1 activity.

## Experimental procedures

### Plant growth conditions and phenotypical characterizations

Nipponbare (*Oryza sativa, ssp. japonica*) and all the transgenic plants used in this study were grown in the experimental field and greenhouse of China National Rice Research Institute. The thousand‐grain‐weight, seed length width and chalkiness of WT and mutant lines were examined by a seed phenotyping system (Wangsheng, Hangzhou, China).

Total starch and amylose contents of brown seedswere measured with a starch assay kits Megazyme K‐TSTA and K‐AMYL (Megazyme, Ireland, UK). The content is expressed as the percentage of total sample weight on an oven‐dry basis. The total amylose, crude fiber, lipid and protein contents in the grains were measured following the previous report (Kang *et al*., 2005). RVA analysis was done on a Rapid Visco Analyzer (RVA Techmaster, Newport Scientific, Narrabeen, Australia) as described by Sun *et al*., 2017. DSC assay was conducted on a differential scanning calorimeter DSC1 STARe system (Mettler Toledo, Switzerland) by following Sun *et al*., 2017.

### RNA isolation, qRT‐PCR and mRNA *in situ* hybridization

The RNA of all the tissues except developing seeds was extracted by Trizol (Invitrogen, Carlsbad, CA). The extraction of developing seed RNA was conducted using a modified SDS‐Trizol method (Qiu *et al*., 2016). RNA reverse transcrition and qRT‐PCR were performed with technical triplicates as described by (Hou *et al*., 2015). These data are presented as mean ± SD. The relative expression level of the tested genes was normalized to *ubiquitin* and calculated by the 2^−ΔΔCT^ method. The mRNA *in situ* hybridization were conducted as described by Zhang *et al*. (2010).

### Vector construction and plant transformation

The CRISPR/Cas9 system was adopted from a previous report (Ma *et al*., 2015). Annealed double strand oligos of the gDNA sequences were ligated into the pYLgRNA‐OsU3 using BsaI site (Thermo, Waltham, MA). gDNA sequences are shown in Table S3. For the overexpression construct of *NF‐YB1*, the CDS was ligated into pU1301 to be driven by a maize ubiquitin promoter (Zhang *et al*., 2010).

The rice variety ‘Nipponbare’ was used as the recipient. *Agrobacterium* strain EHA105 was used for transformation. *Agrobacterium*‐mediated transformation was conducted as described by Hiei *et al*., 1994.

### Bacterial‐two‐hybrid assay

Fragments of *NF‐YB1* and *NF‐YC8‐12* were cloned into pBT and pTRG vectors using the enzyme sites as indicated in Table S3. BacterioMatch II Two‐Hybrid System was purchased from Agilent Technology (San Francisco, CA). Plasmids of baits and preys were co‐transformed into *E. coli* strain XL1‐Blue MRF′ *via* heat shock, and the colonies were selected on M9/+Cm/+Ter/(M9 medium containing 34 μg/mL chloramphenicol, 12.5 μg/mL tetracycline) and M9/+Cm/+Ter/+3‐AT/+Strep/‐HIS mediums (M9 medium containing 34 μg/mL chloramphenicol, 12.5 μg/mL tetracycline, 5 mm 3‐amino‐1,2,4‐triazole, 12.5 μg/mL streptomycin and histidine dropped out) respectively. LGF2‐pBT and Gal11‐pTRG provided in the kit were used as CK+, pBT and pTRG empty vector was used as CK‐.

### Purification of tag‐fused proteins and *in vitro* pull‐down assay

For the recombinant protein expression, the CDS of *NF‐YB1* and *NF‐YC12* were amplified and cloned into pET28a (Merck, Darmstadt, Germany) and pGEX‐4T‐1 (GE Healthcare, Chicago, IL) respectively. For *HIS‐bHLH144‐FLAG* construct*,* FLAG sequence was synthesized on the reverse primer, and then the amplicon was cloned into the pET28a (Merck, Darmstadt, Germany).

HIS‐NF‐YB1, GST‐NF‐YC12, HIS‐bHLH144‐FLAG recombinant proteins were induced in *E. coli* strain *Rossetta*, and purified by Glutathione‐Sepharose Resin Protein Purification Kit and 6 X His‐Tagged Protein Purification Kit (CWBIO, Beijing, China) respectively. Pull‐down assay was conducted as following: 50 μL equilibrated Glutathione High Capacity Magnetic Agarose Beads (Sigma, St Louis, MO) or anti‐FLAG M2 Magnetic beads (Cat No. M8823, Sigma‐Aldrich, St. Louis, MO) was mixed with 500 μg of each recombinant protein in 600 μL pull‐down buffer (50 mm Tris‐HCl pH = 7.5, 5% glycerol, 1 mm EDTA, 1 mm DTT, 1 mm PMSF, 0.01% Nonidet P‐40, 150 mm KCl) under 4 °C for 2 h. The bound proteins together with the beads were collected, washed with pull‐down buffer twice, eluted with 50 μL 1× PBS and immune detected by GST (Cat: CW0085, CWBIO, Beijing, China), HIS (Cat: CW0083, CWBIO, Beijing, China) and FLAG (Cat: CW0287, CWBIO, Beijing, China) antibodies respectively.

### Yeast‐three‐hybrid assay

NF‐YB1 CDS was cloned to fuse with GAL4 BD domain, and NF‐YC12 was driven by a methionine responsive promoter *Met25* in pBRIDGE (Clontech, Dalian, China). NF‐YB1‐NF‐YC12‐pBRIDGE in strain Y2HGold was mated with an AD domain‐fused seed cDNA library in Y187 strain. The mated transformants were first selected on SD/‐Leu/‐Trp/‐His/‐Ade/‐Met. Positive colonies were then transferred to SD/‐Leu/‐Trp/‐His/‐Ade/‐Met/+X‐α‐Gal and SD/‐Leu/‐Trp/‐His/‐Ade/+X‐α‐Gal respectively. The interaction was confirmed by the visualization of blue colonies on the medium.

### BiFC assay

We cloned NF‐YB1 CDS into MCS1 of pDOE‐BiFC vector for N‐terminal fusions to create a ‘parent vector’ and NF‐YC12 and bHLH‐144 were cloned into MCS3 site for C‐terminal fusions (Gookin and Assmann, 2014). In addition, full CDS except stop codon of NF‐YC12 and bHLH144 were cloned into pCAMBIA1305‐mcherry. Constructs were electroporated into Agrobacterium strain EHA105 and subsequently infiltrated into the leaf epidermal cells of 3‐week‐old *Nicotiana benthamiana*. Confocal microscopy was performed using a Zeiss LSM710 confocal laser scanning microscopy (Carl Zeiss AG, Jena, Germany) at 72 h after infiltration.

### Yeast‐one‐hybrid assay

The Clontech™ one‐Hybrid System (Clontech, Dalian, China) was used in this study. The CDS of potential transactivators were fused with GAL4 AD domain in pB42AD (Clontech, Dalian, China), and the promoter region of *Wx* were cloned into pLacZ2u (Clontech, Dalian, China). Yeast strain EGY48 was transformed with indicated plasmids and grew on SD/‐Ura/‐Trp plates, and then strike on SD/‐Ura/‐Trp plates containing 2% glactose, 1% raffinose, 1 × BU salts and 80 mg/L X‐Gal (Clontech, Dalian, China). The interaction was confirmed by the visualization of blue colonies on the medium. NF‐YB1‐pB42AD and SUT4‐pLacZ2u were used as CK+ (Bai *et al*., 2015). The empty vectors pLacZ2u and pB42AD were used as negative control.

### Luciferase transient transcriptional activity assay

The CDS of *NF‐YB1*,* NF‐YC12* and *bHLH144* were, respectively, cloned into ‘None’ vector as effectors and the promoter region of *Wx* was cloned into 190LUV vector as reporter (Zong *et al*., 2016). Protoplast preparation and transformation were conducted according to the method of (Xie and Yang, 2013). Luciferase^®^ Reporter Assay System (Promega, Madison, WI) was used to measure the luciferase activity according to manufacturer instruction. The relative luciferase activity was calculated as the ratio between rLUS1 and rLUS2.

### Electrophoresis mobility shift assay

Electrophoresis mobility shift assay probes in a length of 59 nt were commercially synthesized by Tsinke Biological Technology (Hangzhou, China) and labeled with an EMSA Probe Biotin Labeling Kit (Cat No. GS008, Beyotime, Shanghai, China). DNA binding was performed in a 10 μL reaction volume containing EMSA/Gel‐shift binding buffer (Beyotime, Shanghai, China), 2 nmol biotin‐labeled probe, 5 nmol purified recombinant protein. Non‐labeled DNA oligos were used as competitor. Recombinant protein was pre‐incubated with the EMSA/Gel‐shift binding buffer for 20 min at 25 °C prior to the addition of the biotin‐labeled probe and further incubated at 25 °C for 20 min. A 6% (W/V) polyacrylamide gel was pre‐run for 30 min, and then the binding reaction is subjected to gel electrophoresis. The DNA probes were then transferred to a charged nylon membrane (Beyotime, Shanghai, China), detected by streptovidin‐HRP (Beyotime, Shanghai, China), and finally visualized using the enhanced chemiluminescence (Pierce, Waltham, MA).

### Chromatin immuno‐precipitation (ChIP) and ChIP‐PCR

Chromatin immuno‐precipitation was performed as described previously (Hou *et al*., 2015). Briefly, chromatin was isolated from 2 to 4 g leaves of *proUbi:NF‐YB1‐FLAG* plants, then fragmented to 200–700 bp by sonication. The DNA/protein complex was immune‐precipitated with ChIP‐grade antibody against FLAG (F1804, Sigma‐Aldrich, St. Louis, MO). After reverse cross‐linking and protease K treatment, the immune‐precipitated DNA was purified. The immune‐precipitated and input DNA was as template for quantitative PCR using gene specific primers (Table S3). The quantitative PCR results were analysed by following a method of Magna ChIP™ HiSens kit (Millipore, MA). All the quantitative ChIP‐PCR was performed in three biological replicates.

### Cell‐free degradation assay

The experiment was conducted by following Lv *et al*., 2014. Briefly, total protein of 10 days‐after‐germination rice seedlings were extracted in degradation buffer (25 mm Tris‐HCl, pH 7.5, 10 mm NaCl, 10 mm MgCl_2_, 4 mm PMSF, 5 mm DTT, and 10 mm ATP) and quantified using a Quabit system (Invitrogen, Carlsbad, CA). Purified recombinant proteins (5 μg of each) were incubated with 200 μg extracted total proteins in 20 μL degradation buffer at 28 °C. Reactions were terminated at indicated time points, and the protein abundance was visualized by immune detection against anti‐HIS. The immune signals were quantified using Quantity Tools of Image Lab software (Bio‐Rad, Hercules, CA). The half‐life of HIS‐NF‐YB1 was calculated based on the degradation curves deduced from the tested time points. The protein intensities were quantified using ImageJ software with triplicates. The dissociation‐one phase exponential decay curve was plotted on a semilog graph using Graphpad Prism (5.0) software as previously described by (Lv *et al*., 2014).

## Author contributions

J.Zhang. planned and designed the research; B.B., Y.H., G.J., Y.W. J.Zhao, Z.L. and X.W. performed experiments; B.B., Y.H., Y.W., X.T. W.Y. and J.Zhang. analysed data; and B.B., and J.Zhang. wrote the manuscript. B.B. and Y.H. contributed equally.

## Supporting information


**Figure S1** Subcellular localization of NF‐YB1.
**Figure S2** Sanger sequencing of the mutated sites in homozygous mutants of *crnf‐yb1*,* crnf‐yc12* and *crbhlh144*.
**Figure S3** Grain weights of *crnf‐yb1s* T2 lines.
**Figure S4** Expression level and grain size of *NF‐YB1* over‐expression lines.
**Figure S5** Y3H assay of the NF‐YB1‐YC12 complex with three seed‐specific NF‐YAs.
**Figure S6** Venn diagram showing the number of co‐regulated DEGs by *NF‐YB1* and *NF‐YC12* as revealed by RNA‐seqs.
**Figure S7** KEGG pathway enrichment analysis of DEGs co‐regulated by *NF‐YB1* and *NF‐YC12*.
**Figure S8** EMSA assay showing the binding of NF‐YB1 to the *Wx* promoter.
**Figure S9** Seed phenotype and genotype of *wx* mutants in the background of Ningjin 7 and Huazhang.

**Table S1** Major agronomic traits of *crnf‐yb1s*,* crnf‐yc12s* and *crbhlh144s*.
**Table S2** Differential scanning calorimetry assay of *crnf‐yb1s*.

**Table S3** Sequences of primers used in this study.

**Table S4** DEGs regulated by *NF‐YB1* and/or *NF‐YC12*.
