# Supplementary material for: NF‐YB1‐YC12‐bHLH144 complex directly activates Wx to regulate grain quality in rice (Oryza sativa L.)
Source: Plant Biotechnol J. 2019 Jan 4;17(7):1222–35. doi: 10.1111/pbi.13048 (PMC6576074; doi:10.1111/pbi.13048)
Supplement: Supplementary file 1 — Figure S1 Subcellular localization of NF‐YB1. Figure S2 Sanger sequencing of the mutated sites in homozygous mutants of crnf‐yb1, crnf‐yc12 and crbhlh144. Figure S3 Grain weights of crnf‐yb1s T2 lines. Figure S4 Expression level and grain size of NF‐YB1 over‐expression lines. Figure S5 Y3H assay of the NF‐YB1‐YC12 complex with three seed‐specific NF‐YAs. Figure S6 Venn diagram showing the number of co‐regulated DEGs by NF‐YB1 and NF‐YC12 as revealed by RNA‐seqs. Figure S7 KEGG pathway enrichment analysis of DEGs co‐regulated by NF‐YB1 and NF‐YC12. Figure S8 EMSA assay showing the binding of NF‐YB1 to the Wx promoter. Figure S9 Seed phenotype and genotype of wx mutants in the background of Ningjin 7 and Huazhang. [file PBI-17-1222-s003.docx]

**Supporting figures**


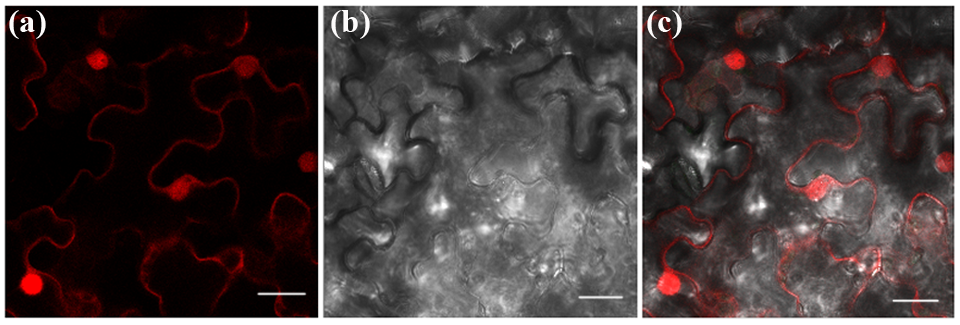


**Fig S1. Subcellular localization of NF-YB1.**

Con-focal microscopy analysis of NF-YB1-RFP in tobacco cells under fluorescence (a), bright field (b) and merged (c).


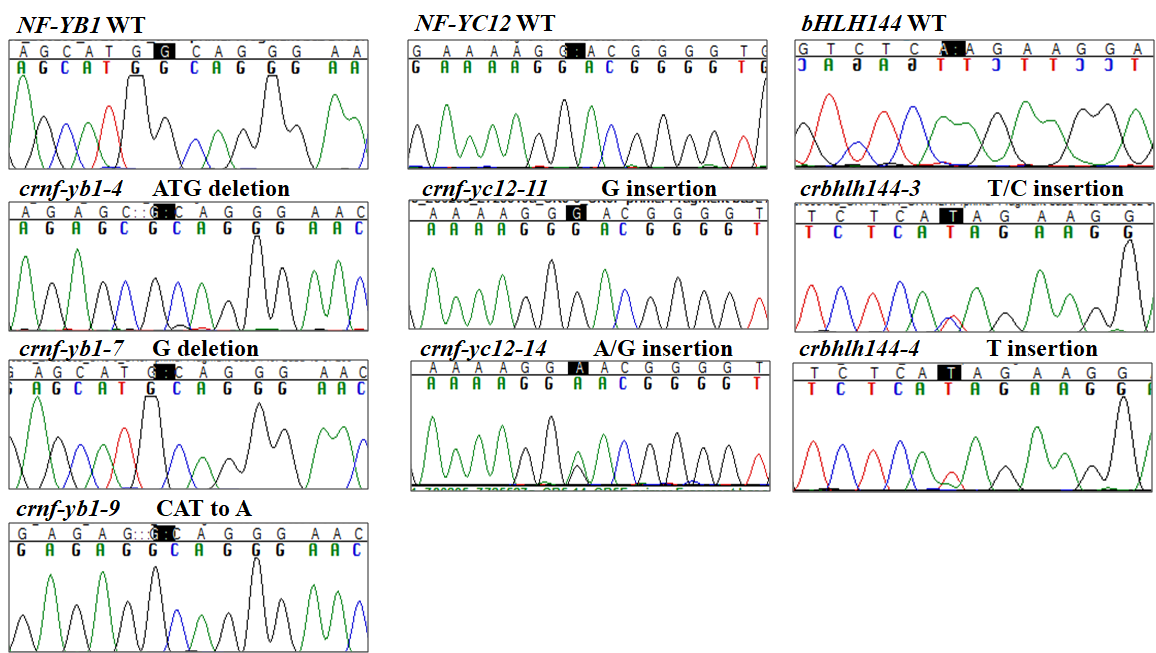


**Fig S2. Sanger sequencing of the mutated sites in homozygous mutants of *crnf-yb1*, *crnf-yc12* and *crbhlh144*.**


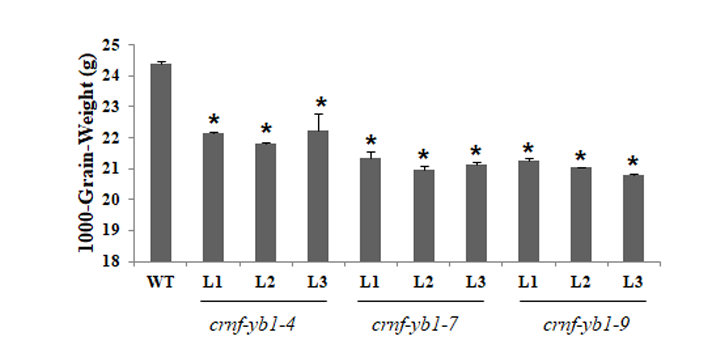


**Fig S3. Grain weights of *crnf-yb1s* T2 lines.**

Data are shown as mean ± SD with triplicates. The asterisk represents significant difference with the WT at P≤0.05 as determined by the Student’s *t* test.


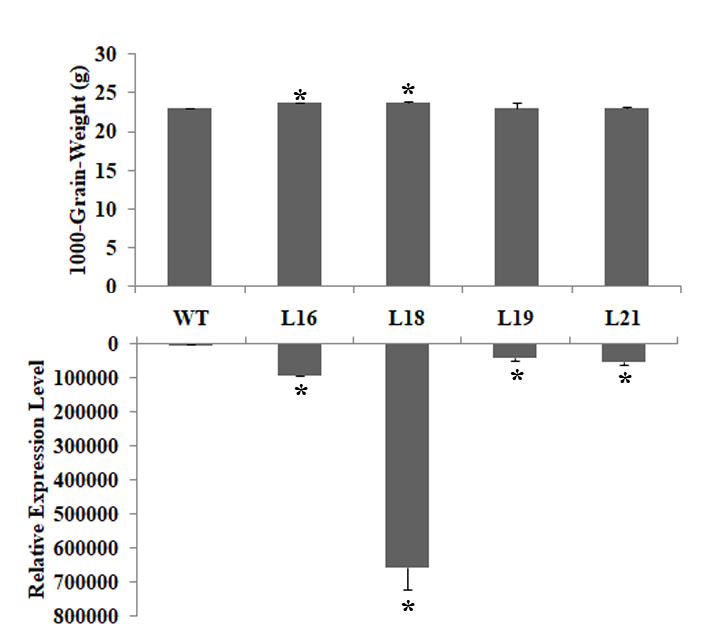


**Fig S4. Expression level and grain size of *NF-YB1* over-expression lines.**

Data are shown as mean ± SD with triplicates. The asterisk represents significant difference with the WT at P≤0.05 as determined by the Student’s *t* test. Leaf cDNAs of T_0_ lines were used for the qRT-PCR analysis of *NF-YB1* level. The relative expression levels were normalized by setting the value of WT to 1. It should be noted that *NF-YB1* is extremely lowly or not expressed in WT leaf.

**
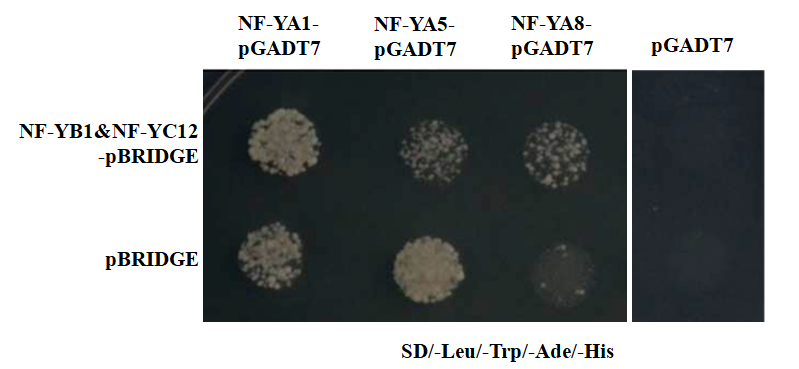
**

**Fig S5. Y3H assay of the NF-YB1-YC12 complex with three seed-specific NF-YAs.**

**
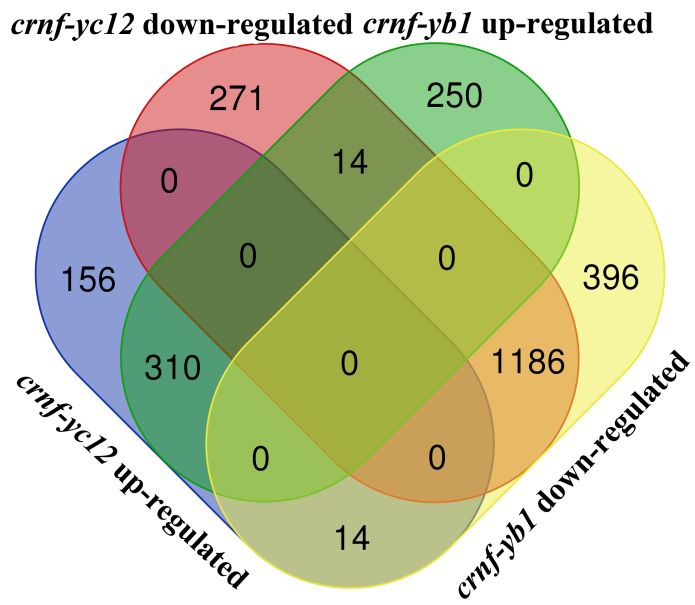
**

**Fig S6. Venn diagram showing the number of co-regulated DEGs by *NF-YB1* and *NF-YC12* as revealed by RNA-seqs.**

**
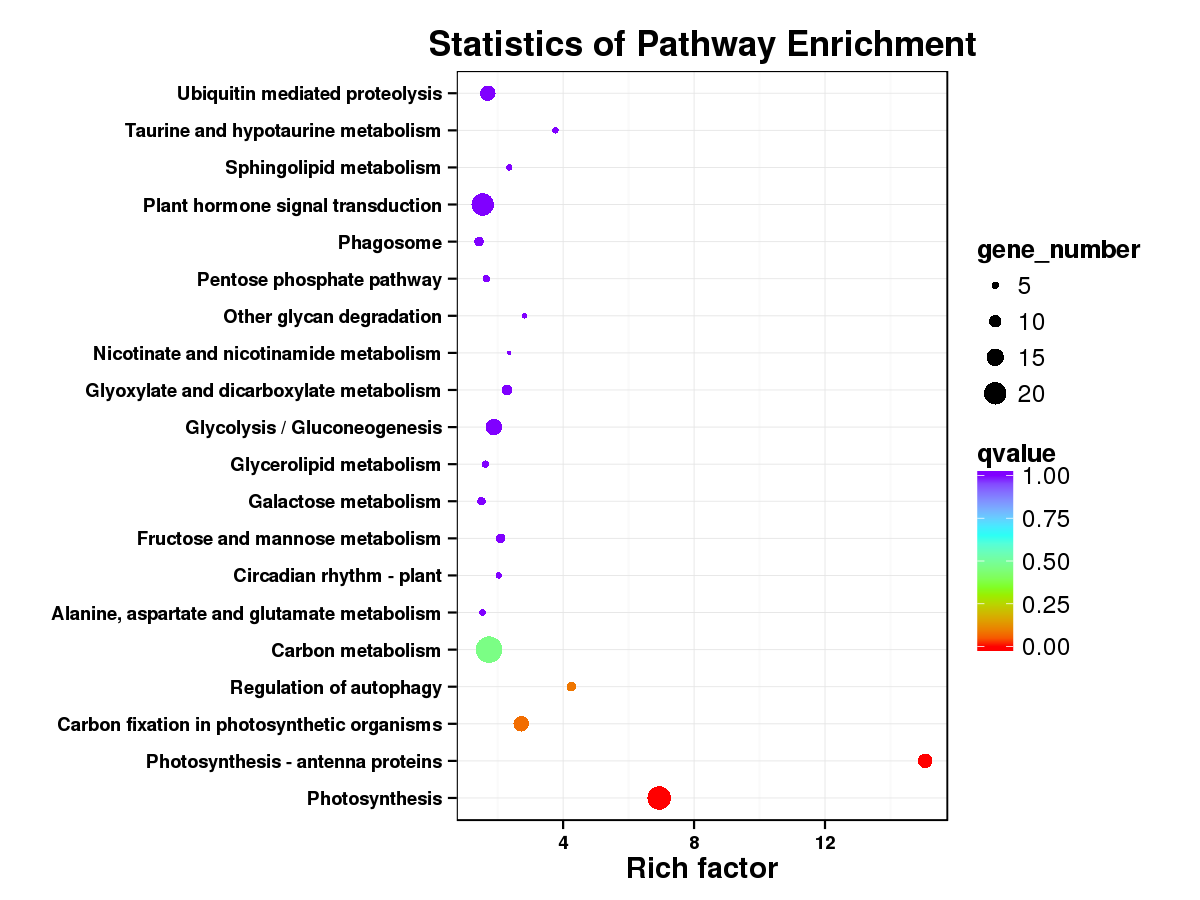
**

**Fig S7. KEGG pathway enrichment analysis of DEGs co-regulated by *NF-YB1* and *NF-YC12*.**


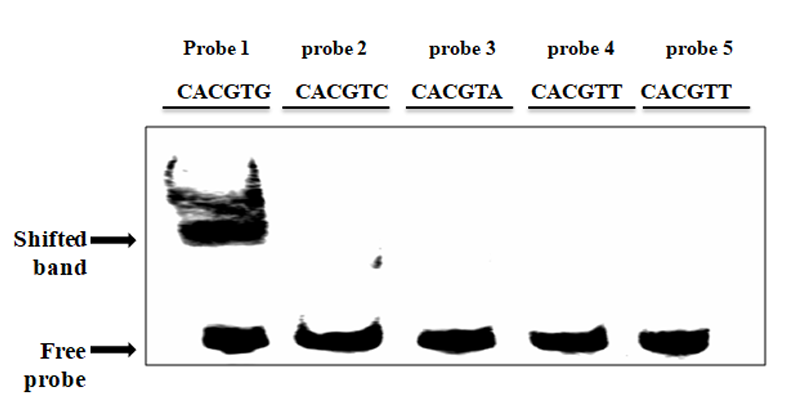


**Fig S8. EMSA assay showing the binding of NF-YB1 to the *Wx* promoter.**

The positions of probe 1-5 can be referred on Fig 5c.


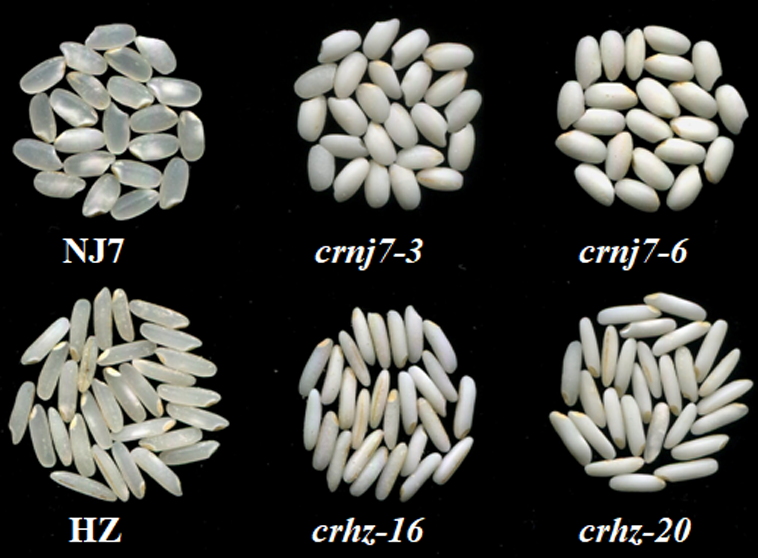


**Fig S9. Seed phenotype of *wx* mutants in the background of Ningjin 7 and Huazhang.**

Milled seeds of homozygous *wx* mutants in Ningjin 7 (*crnj7*) and Huazhang (*crhz*) from T_0_ plants.
