# Supplementary material for: NF‐YB1‐YC12‐bHLH144 complex directly activates Wx to regulate grain quality in rice (Oryza sativa L.)
Source: Plant Biotechnol J. 2019 Jan 4;17(7):1222–35. doi: 10.1111/pbi.13048 (PMC6576074; doi:10.1111/pbi.13048)
Supplement: Supplementary file 2 — Table S1 Major agronomic traits of crnf‐yb1s, crnf‐yc12s and crbhlh144s. Table S2 Differential scanning calorimetry assay of crnf‐yb1s. [file PBI-17-1222-s002.docx]

**Table S1. Major agronomic traits of *crnf-yb1s*, *crnf-yc12s* and *crbhlh144s*.**

|  | Plant height (cm) | Flowering date (days) | Seed setting (%) | Seed length (mm) | Seed width (mm) | 1000-grain-weight (g) |
| --- | --- | --- | --- | --- | --- | --- |
| WT | 72.07±3.16 | 71.67±1.25 | 88.38±1.65 | 7.90±0.05 | 3.35±0.04 | 25.58±0.12 |
| *crnf-yb1-4* | 70.40±1.44 | 74.33±2.05 | 87.79±1.58 | 7.60±0.05* | 3.23±0.04* | 22.87±0.06* |
| *crnf-yb1-7* | 70.43±2.15 | 73.00±1.63 | 87.65±1.56 | 7.66±0.04* | 3.24±0.02* | 21.58±0.16* |
| *crnf-yb1-9* | 70.50±1.39 | 75.00±2.45 | 88.02±1.62 | 7.55±0.01* | 3.25±0.02* | 20.98±0.20* |
| *crnf-yc12-7* | 70.77±1.27 | 73.00±2.94 | 87.47±1.47 | 7.78±0.04* | 3.23±0.05* | 21.37±1.07* |
| *crnf-yc12-11* | 69.87±1.99 | 73.67±1.25 | 87.37±1.83 | 7.72±0.05* | 3.25±0.02* | 20.01±0.56* |
| *crnf-yc12-14* | 68.40±1.65 | 73.33±2.05 | 87.58±1.77 | 7.72±0.03* | 3.25±0.03* | 18.96±0.08* |
| *crbhlh144-2* | 72.67±1.69 | 69.66±1.69 | 87.46±181 | 7.96±0.09 | 3.37±0.03 | 25.46±0.33 |
| *crbhlh144-3* | 74.97±4.49 | 69.00±1.63 | 87.29±1.80 | 7.91±0.04 | 3.55±0.03* | 25.79±0.16 |
| *crbhlh144-4* | 77.57±4.63 | 69.00±1.63 | 87.57±1.21 | 8.05±0.03* | 3.23±0.03* | 25.12±0.25 |

The data is presented as the Means ± SD of at least three biological replicates. The asterisks represent significant difference between the WT and mutants as determined by the Student’s *t* test, the single asterisk indicates P≤0.05, double asterisks indicate P≤0.01.

**Table S2. Differential scanning calorimetry assay of *crnf-yb1s*.**

|  | Onset gelatinization T（℃） | Peak gelatinization T（℃） | End gelatinization T（℃） | Gelatinization enthalpy (J/g) |
| --- | --- | --- | --- | --- |
| WT | 67.54±0.13 | 73.19±0.03 | 79.89±0.14 | 7.09±0.11 |
| *crnf-yb1-4* | 65.59±0.09^**^ | 71.59±0.09** | 78.53±0.12** | 6.67±0.17** |
| *crnf-yb1-7* | 62.62±0.08** | 68.83±0.19** | 78.75±0.02** | 6.56±0.08** |
| *crnf-yb1-9* | 62.46±0.12** | 68.61±0.11** | 75.56±0.15** | 6.15±0.03** |

Brown seeds were used for the assays. The data is presented as the Means ± SD of at least three biological replicates. The asterisks represent significant difference between the WT and mutants as determined by the Student’s *t* test, the single asterisk indicates P≤0.05, double asterisks indicate P≤0.01.
